# Who Should Be Responsible for Its Support?

**Published:** 1912-06-08

**Authors:** 


### THE IDEAL SCHOOL CLINIC.

### Who Should be Responsible for its Support?

Very naturally one of the first questions which arises when the subject of the establishment of a school clinic is under consideration is who should pay for the upkeep of such a centre of treatment. With the example of the voluntary system, as applied to hospitals and other means for the cure and relief of sickness, before us, it is equally natural that there should be a desire on the part of a section of the public to see such treatment centres established and kept up by private munificence. On the other hand, there is a growing feeling that school clinics should be, like the schools themselves, paid for by the citizens from the general taxes so that the burden of their upkeep is equitably distributed.

### The Criticisms of School Doctors.

The voluntary clinic is at present the rule in England. The clinics at Croydon, Hampstead, Wandsworth, and Deptford are all voluntary clinics. They are all open to the same objections which may be urged against centres of treatment which are subject to no official control beyond that of mere approval resulting from whatever good results may be obtained. While we fully admit the good work that has been carried on by these centres of treatment, no one who has had any practical experience of the manner in which that work is carried out can be thoroughly satisfied that the voluntary clinics are the best that can be obtained. We may even go further and say that few school doctors will be satisfied that these clinics, notwithstanding the fact that they do a certain amount of good, are wholly desirable in view of the fact that the results of treatment are so varying and, on the whole, so indifferent. Some of the reasons for this diversity of treatment we have already outlined in previous articles; the chief reason, probably, is that the voluntary clinic is staffed by local practitioners who do not possess the requisite experience and skill to deal with the specialities they are forced to undertake. Instances of the manner in which the work is being shirked and wrongly done have already been given; it is only necessary to repeat here that no voluntary clinic which has yet been established in this country is wholly satisfactory, either in regard to treatment or diagnosis.

In favour of the voluntary system, as applied to school clinics, it is stated that the average cost per child in voluntary is lower than that in State or municipal clinics. This assertion is made on insufficient evidence. Indeed, when we tabulate the available returns of income and outlay of the voluntary clinics and the municipal-aided treatment centres, it is easy to demonstrate that the cost per head of the latter is appreciably lower than that of the former. When we gauge costliness of treatment by the effective results of such treatment—naturally a somewhat difficult method of comparison, which is only applicable in a certain number of cases—we will probably find that the advantage is very much in favour of the rate-aided centre. The staff of the voluntary centre has to work under what may legitimately be called adverse economic conditions, with the result that there is little uniformity in the cost of treatment. We see the same want of uniformity as regards cost of treatment in small hospitals or in dispensaries staffed by practitioners who do not undertake the treatment of special cases, such as orthopædic defect, etc., as a matter of routine; it arises solely from a want of experience, which again conduces to extravagance in the employment of materials such as drugs, dressings, etc.

### Who Should Form the Staff?

Yet another point in favour of the voluntary clinic, it is alleged, is the fact that it raises no opposition from the profession resident in its immediate neighbourhood. This argument it is scarcely necessary to notice in detail. Whether a school clinic does or does not obtain the support and encouragement of the practitioners who, while residing in the neighbourhood, have no active participation in its work, depends entirely on the manner in which it is managed. No policy can be worse calculated to further the interests of the children, and consequently of the public, than that of attempting to placate local practitioners by appointing to the staff men who are utterly incapable of doing the special work required except under close supervision. Indeed, it is justifiable to argue that a voluntary clinic does more harm to the practitioners resident in the neighbourhood than a rate-aided clinic manned by specialists. It is absurd to make all the local practitioners members of its staff; there must necessarily be some exclusion and selection, and these by themselves will affect the interests of the local profession. This point also we have elaborated in a former article, and it is one which is worth some consideration. A voluntary clinic will be in almost exactly the same position as a voluntary hospital—and experience has shown that the latter can be very unpopular with local practitioners if it is managed in a way that disregards their interests and flouts their legitimate wishes. At the same time this argument cannot be counted as being of great value. The profession is gradually recognising, unpalatable as the process of recognition seems, that it is no longer a close corporation to which everything must be subservient, but that it is concerned with the health of the community—a matter in which the State has as much interest as the profession. The idea of a State medical service is gradually dawning upon many, and with it the corollary that it is unfair to enact compulsory education without free medical treatment of defective school children, where such is imperatively required.

That being the case, the voluntary clinic must be regarded as only a temporary convenience which is bound, in course of time, to be assimilated with a rate-supported institution as has been the case abroad.

